# Complement and the Alternative Pathway Play an Important Role in LPS/D-GalN-Induced Fulminant Hepatic Failure

**DOI:** 10.1371/journal.pone.0026838

**Published:** 2011-11-01

**Authors:** Shihui Sun, Yan Guo, Guangyu Zhao, Xiaojun Zhou, Junfeng Li, Jingya Hu, Hong Yu, Yu Chen, Hongbin Song, Fei Qiao, Guilian Xu, Fei Yang, Yuzhang Wu, Stephen Tomlinson, Zhongping Duan, Yusen Zhou

**Affiliations:** 1 State Key Laboratory of Pathogen and Biosecurity, Beijing Institute of Microbiology and Epidemiology, Beijing, China; 2 Beijing You-An Hospital, Artificial Liver Center, Capital University of Medical Sciences, Beijing, China; 3 Institute of Disease Control and Prevention, Academy of Military Medical Science, Beijing, China; 4 Department of Microbiology and Immunology, Medical University of South Carolina, Charleston, South Carolina, United States of America; 5 Institute of Immunology, College of Basic Medical Sciences, Third Military Medical University, Chongqing, China; National Institute of Environmental Health Sciences, United States of America

## Abstract

Fulminant hepatic failure (FHF) is a clinically severe type of liver injury with an extremely high mortality rate. Although the pathological mechanisms of FHF are not well understood, evidence suggests that the complement system is involved in the pathogenesis of a variety of liver disorders. In the present study, to investigate the role of complement in FHF, we examined groups of mice following intraperitoneal injection of LPS/D-GalN: wild-type C57BL/6 mice, wild-type mice treated with a C3aR antagonist, C5aR monoclonal antibody (C5aRmAb) or CR2-Factor H (CR2-fH, an inhibitor of the alternative pathway), and C3 deficient mice (C3^−/−^ mice). The animals were euthanized and samples analyzed at specific times after LPS/D-GalN injection. The results show that intraperitoneal administration of LPS/D-GalN activated the complement pathway, as evidenced by the hepatic deposition of C3 and C5b-9 and elevated serum levels of the complement activation product C3a, the level of which was associated with the severity of the liver damage. C3a receptor (C3aR) and C5a receptor (C5aR) expression was also upregulated. Compared with wild-type mice, C3^−/−^ mice survived significantly longer and displayed reduced liver inflammation and attenuated pathological damage following LPS/D-GalN injection. Similar levels of protection were seen in mice treated with C3aR antagonist,C5aRmAb or CR2-fH. These data indicate an important role for the C3a and C5a generated by the alternative pathway in LPS/D-GalN-induced FHF. The data further suggest that complement inhibition may be an effective strategy for the adjunctive treatment of fulminant hepatic failure.

## Introduction

Fulminant hepatic failure (FHF) is a severe clinical syndrome characterized by hepatic cell injury resulting from a variety of hepatic disease processes, leading to multiorgan failure [1], [2]. Although the incidence of FHF is low, the associated mortality is extremely high and is always related to liver transplantation, viral infection and shock [3]. Bacterial lipopolysaccharide (LPS), the main pathogenic component of gram-negative bacteria, can cause systemic inflammatory response syndrome, which may lead to acute liver injury and multiorgan failure. D-galactosamine (D-GalN) increases the sensitivity of mice to LPS and augments the lethal effects of LPS [4], [5]. Mouse models of LPS/D-GalN-induced hepatitis have been previously described [6], [7]. It has been reported that tumor necrosis factor (TNF)-α-mediated hepatocyte apoptosis may be the cause of LPS-induced liver injury [8]–[10].

The complement system plays important roles in mediating both acquired and innate responses against microbial infection and in immune homeostatic processes, such as the removal of immune complexes and apoptotic cells [11]. Recent evidence from several studies has suggested that the complement system is involved in the pathogenesis of a variety of liver disorders, including liver fibrosis, viral hepatitis, alcoholic liver disease and hepatic ischemia/reperfusion injury (IRI) [12]–[16]. In these disease settings, complement activation products promote tissue inflammation and injury, particularly via the generation of the complement activation products C3a and C5a, which promote inflammation via direct and indirect mechanisms by interacting with their receptors [17]–[19].

Although complement activation has been reported in LPS-treated liver and lung tissues [20]–[22], little is known about the role of complement in FHF, especially during the early period of the disease. In this study, the role of complement in fulminant hepatic failure was systematically investigated using the LPS/D-GalN-induced FHF mouse model. Our study further analyzed the important role of alternative pathway-generated C3a in LPS/D-GalN-induced FHF and suggested a promising strategy for the adjunctive clinical treatment of fulminant hepatic failure.

## Materials and Methods

### Ethics statement

All procedures involving animals were approved by the Laboratory Animal Center, State Key Laboratory of Pathogen and Biosecurity, Beijing Institute of Microbiology and Epidemiology IACUC's (The permitted number is BIME 2009-15). The study of animals was carried out in strict accordance with the recommendations in the Guide for the Care and Use of Laboratory Animals.

### Animals and materials

Wild-type (wt) female C57BL/6 and C3^−/−^ female mice (B6.129S4-C3*^tm1Crr^*/J) 8 weeks of age and weighing 20–25 g were used in this study. Lipopolysaccharide (LPS; from *Escherichia coli* strain 0111:B4) and D-GalN were purchased from Sigma. All drugs were dissolved in pyrogen-free saline. The C3aR antagonist (SB 290157, #559410) was purchased from Calbiochem. Monoclonal antibody to mouse C5aR (HM1076) was purchased from Hycult Biotechnology B.V (Hycult Biotechnology, The Netherlands). CR2-fH was prepared as previously described [23].

### Treatment of mice with LPS/D-GalN

The mice were divided into the following groups: wt mice treated with LPS/D-GalN (wt group), C3^−/−^ mice treated with LPS/D-GalN (C3^−/−^ group), wt mice treated with LPS/D-GalN and C3aR antagonist (C3aR antagonist group), wt mice treated with LPS/D-GalN and C5aRmAb (C5aRmAb group), and wt mice treated with LPS/D-GalN and CR2-fH (CR2-fH group). Wild-type mice treated with saline were used as the control group (0 hour group). The treatment protocols were as follows. The C3aR antagonist (2 mg/kg, diluted in saline containing 0.5% DMSO) and C5aRmAb (600 ug/kg) was injected i.v. 45 min before and 45 min after the LPS/D-GalN injection. CR2-fH (40 mg/kg) dissolved in PBS was injected i.v. immediately after the LPS/D-GalN injection. The dose of C3aR antagonist and CR2-fH used was based on previously reported doses and were in line with doses used in various mouse models of inflammation and injury [24]–[26]. LPS/D-GalN was dissolved in 200 µl saline and injected i.p. at 2.5 µg/kg LPS and 300 mg/kg D-GalN. The animals were euthanized 0, 1, 4 and 8 hours after the LPS/D-GalN injection. Plasma or serum samples were collected for the analysis of C3a, liver enzymes and proinflammatory cytokines. Liver tissues were processed for histopathological analysis and immunostaining to evaluate the C3, C3aR, C5aR and C5a-9 deposition and the expression of *C3aR* mRNA and *C5aR* mRNA.

### Histological analysis of liver damage

The liver tissue was fixed in 10% formalin at room temperature and subsequently embedded in paraffin. Liver sections from each animal were sliced and stained with Hematoxylin and eosin (H&E). The extent of the liver damage was assessed by two independent observers blinded to the treatment groups.

### Measurement of serum C3a levels in mice

To assess whether complement was activated in the liver by LPS/D-GalN, plasma levels of the complement activation product C3a were measured using ELISA (BD Pharmingen). Briefly, a 96-well plate was coated with purified rat anti-mouse C3a antibody (BD Pharmingen, 558250) overnight at 4°C and then blocked with 10% FBS for 1 hour at room temperature. The samples were diluted 1∶2 in blocking buffer and incubated for 2 hours. Bound C3a was detected using a biotinylated rat anti-mouse C3a antibody (BD Pharmingen, 558250) and avidin/horseradish peroxidase(BD Pharmingen, 554058). Reactive C3a levels were measured using the TMB substrate solution (BD Pharmingen 555214).

### Immunostaining to assess complement deposition

Frozen liver sections were sliced and fixed in cold acetone. The sections were incubated overnight at 4°C with rat anti-mouse C3mAb (1∶20 dilution, HyCult Biotechnology bv, Uden, Netherlands), rabbit anti-mouse C3aR polyclonal antibody(1∶50 dilution, Santa Cruz Biotechnology), rabbit anti-mouse C5aR polyclonal antibody (1∶80 dilution, Santa Cruz Biotechnology) and anti-C5b-9 polyclonal antibody (5 ug/ml, Calbiochem,SanDiego,CA). Biotinylated IgG was then added, followed by an avidin-biotin-peroxidase conjugate (Beijing Zhongshan Biotechnology Co., Ltd.). Immunoreactivity was detected using DAB and by counterstaining with hematoxylin.

### Biochemical evaluation of liver injury and proinflammatory cytokine expression in serum

Blood was collected at 0, 1, 4 and 8 hours. Serum samples were prepared as previously described [27] and stored at −80°C. The extent of liver injury was determined by measuring the concentrations of alanine aminotransferase (ALT) and proinflammatory cytokines (IL-6, IL-10, MCP-1, IFN-γ, TNF-α, and IL-12p70) in the serum. ALT was measured using a Beckman CX5 Chemistry Analyzer. The cytokines were assayed using the BD™ CBA Mouse Inflammation Kit (Cat.No. 552364) according to the manufacturer's instructions.

### Detection of *C3aR* mRNA and *C5aR* mRNA by relative quantitative real-time PCR

Total RNA was isolated from the liver tissue using the RNeasy Mini kit (Qiagen) according to the manufacturer's instructions. Any genomic DNA contamination was eliminated by treating the samples with RNase-free DNase (Promega). RT was performed with 1 µg of total RNA, using an RT kit (TaKaRa) according to the manufacturer's instructions. After the reverse transcription step, which comprised incubations for 45 min at 48°C, 5 min at 99°C and 5 min at 5°C, *C3aR* mRNA and *C5aR* mRNA expression levels (normalized to GAPDH) were quantified using SYBR Green real-time PCR (TIANGEN, China) with primers designed by PRIMERS software. The primers for *C3aR* were sense 5′-tctcactgaggcatctattcagtt-3′ and antisense 5′-attgccgtgctacgttctg-3′. The primers for *C5aR* were sense 5′cgctcatcctgctcaacat-3′ and antisense 5′-acggtcggcactaatggtag-3′. The relative *C3aR* and *C5aR* expression data were analyzed using the 2^−ΔΔ*C*T^ method [28].

### Survival analysis

In the survival study, an additional eight mice in each group (the wt, C3^−/−^, C3aR antagonist, C5aRmAb and CR2-fH groups) were monitored for 24 hours period after which all mice were euthanized. Any mice showing signs of severe distress such as weakness, lack of mobility, severe jaundice and presented moribund conditions during this period were euthanized and recorded as end point in survival study. Mice in the event of severe distress were euthanized by overdose of pentobarbital (150 mg/kg, i.p.) followed by cervical dislocation.

### Statistical analysis

The significance of differences between groups was analyzed using a one-way ANOVA and subjected to Tukey's multiple comparisons test. Differences in cytokine and ALT levels between the groups at the indicated times were analyzed using a two-way ANOVA. Survival differences were analyzed using a Kaplan-Meier survival curve with a log-rank test. The data are represented as the mean±SEM. All the analyses were performed using GraphPad Prism software. *P* values lower than 0.05 were considered statistically significant.

## Results

### Complement activation in fulminant hepatic failure induced by LPS/D-GalN

**Figure 1:**
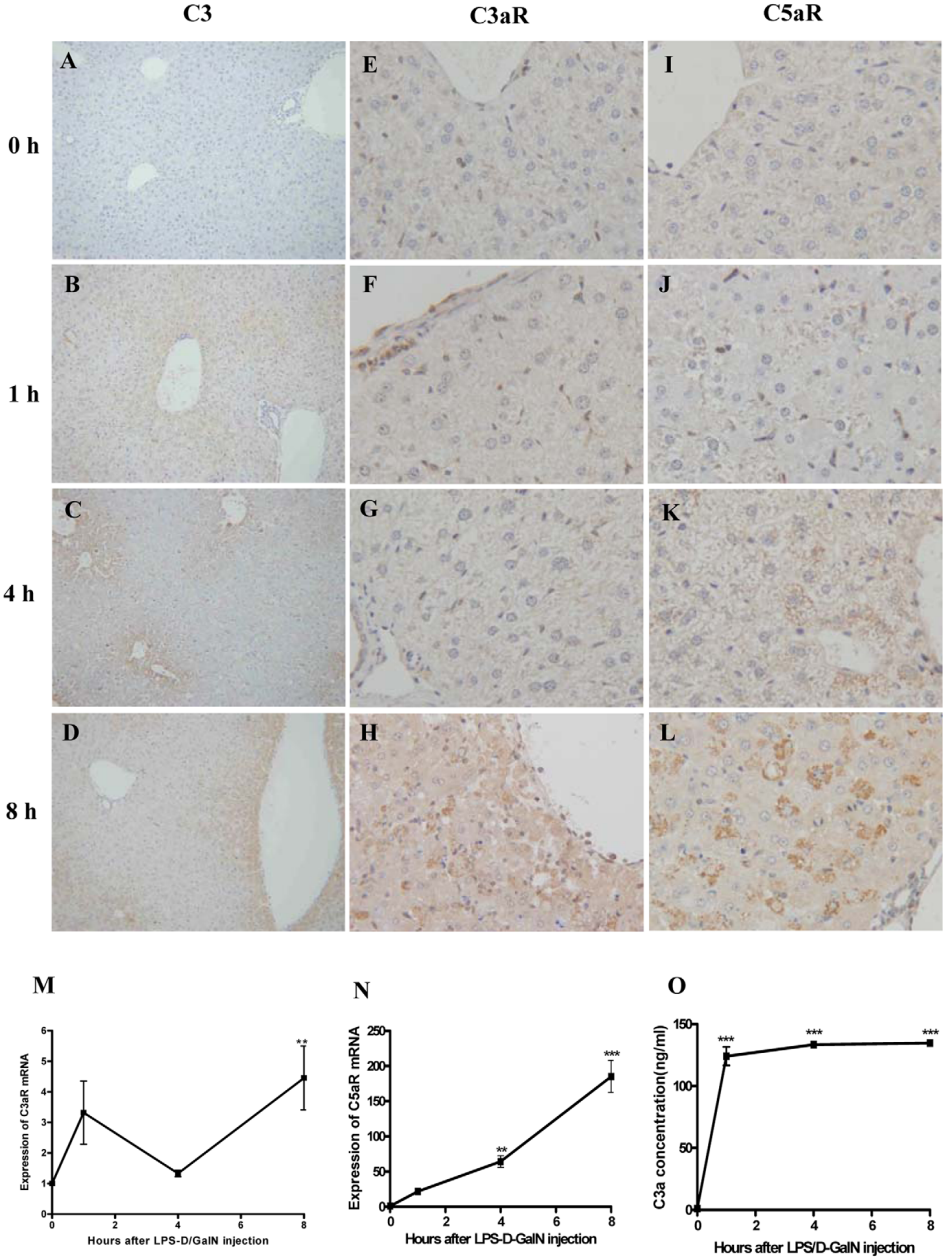
Complement activation following LPS/D-GalN injection. Wt C57BL/6 mice were injected i.p. with LPS/D-GalN and euthanized at 0, 1, 4 and 8 hours after the injection. (A–L) Immunohistochemical staining for C3, C3aR and C5aR in liver sections after the LPS/D-GalN injection. (M–N) The relative *C3aR mRNA* and *C5aR* mRNA expression levels were determined in live tissue at the indicated time points after the LPS/D-GalN injection. The mRNA expression was determined by relative quantitative real-time PCR analysis. The results are expressed as the means±SEM relative to GAPDH expression. (O) The serum concentrations of C3a at the indicated times after the LPS/D-GalN injection. ** and *** indicate *p*<0.01 and *p*<0.001, respectively. n = 6–7 per group. The original magnification for C3 of stained images: ×200; for C3aR and C5aR of stained images: ×800.

C3 was deposited in the liver parenchyma of the LPS/D-GalN-treated mice, as detected by immunohistochemistry. A low level of C3 deposition was detected around the central vein (CV) at the 0-hour time point (Fig. 1A), whereas increased levels of C3 deposition were observed in the parenchyma, especially around the CV, 1 hour after the LPS/D-GalN injection (Fig. 1B), although no obvious pathologic injury was detected. The C3 deposition levels in the liver sections progressively increased over time after the LPS/D-GalN injection, reaching their highest value at 8 hours, primarily in the centrilobular regions (Fig. 1C and D).

Protein and gene expression levels of C3aR and C5aR were analyzed in liver tissue samples isolated at 0, 1, 4 and 8 hours post-LPS/D-GalN injection by immunohistochemistry and relative quantitative real-time RT-PCR. The results show that C3aR and C5aR expressed on non-parenchymal cells, especially kupffer cells in mice without LPS/D-GalN injection. However, the expression of C3aR and C5aR on both non-parenchymal cells and hepatocytes increased with time after LPS/D-GalN injection (Fig. 1E–L). *C3aR* mRNA and *C5aR* mRNA expression levels correlated with immunohistological data. *C3aR* mRNA increased 1 hour after the LPS/D-GalN injection, but we consistently found that *C3aR* mRNA levels returned to near control levels at 4 hours, and then increased again at 8 hours after the LPS/D-GalN injection (*p*<0.01) (Fig. 1M). In contrast, the C5aR expression linearly increased with time throughout the 8 hour period following the LPS/D-GalN injection (Fig. 1N). In addition, the plasma C3a levels were significantly elevated as early as 1 hour after the LPS/D-GalN injection and remained elevated for 8 hours (Fig. 1O). Collectively, the above data demonstrate significant complement activation in the liver post-LPS/D-GalN injection, and the extent of complement activation correlated with the severity of the liver injury.

### C3 deficiency attenuates LPS/D-GalN-induced pathologic damage in the liver

**Figure 2:**
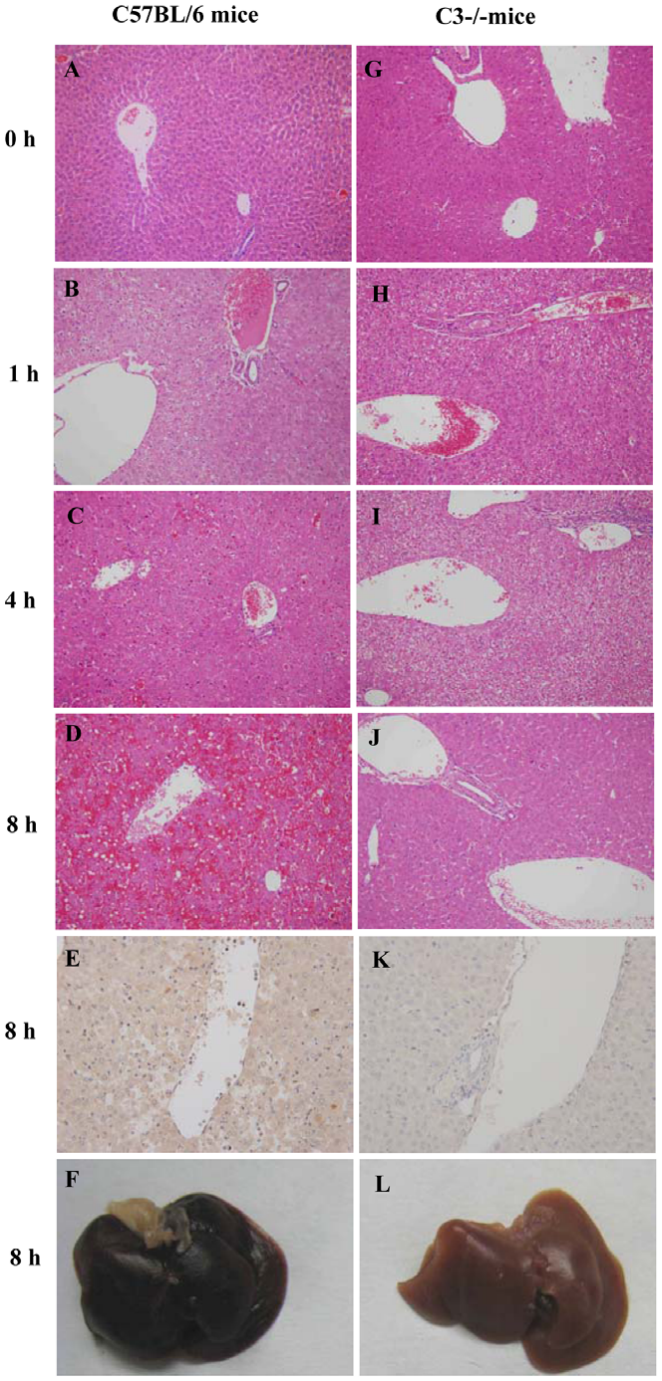
C3 deficiency alleviated liver injury after the LPS/D-GalN injection. Wt C57BL/6 mice and C3^−/−^ mice were injected with LPS/D-GalN i.p. and euthanized at 0, 1, 4 and 8 hours after the LPS/D-GalN injection. The left panels (A–D) shows the H&E staining of wt C57BL/6 mouse liver sections at the designated time points and demonstrates the progression of liver damage over time. The right panels (G–J) shows the corresponding liver sections of the C3^−/−^ mice, revealing less severe liver damage. (E, K) Immunohistochemical staining for C5b-9 in liver sections in wt mice and C3^−/−^ mice 8 hours after LPS/D-GalN injection. (F, L) Depiction of whole explanted livers demonstrating the different degree of hemorrhage. Each image is representative of 4–5 mice per group. Magnification of the H&E stained images: ×200.

**Figure 3:**
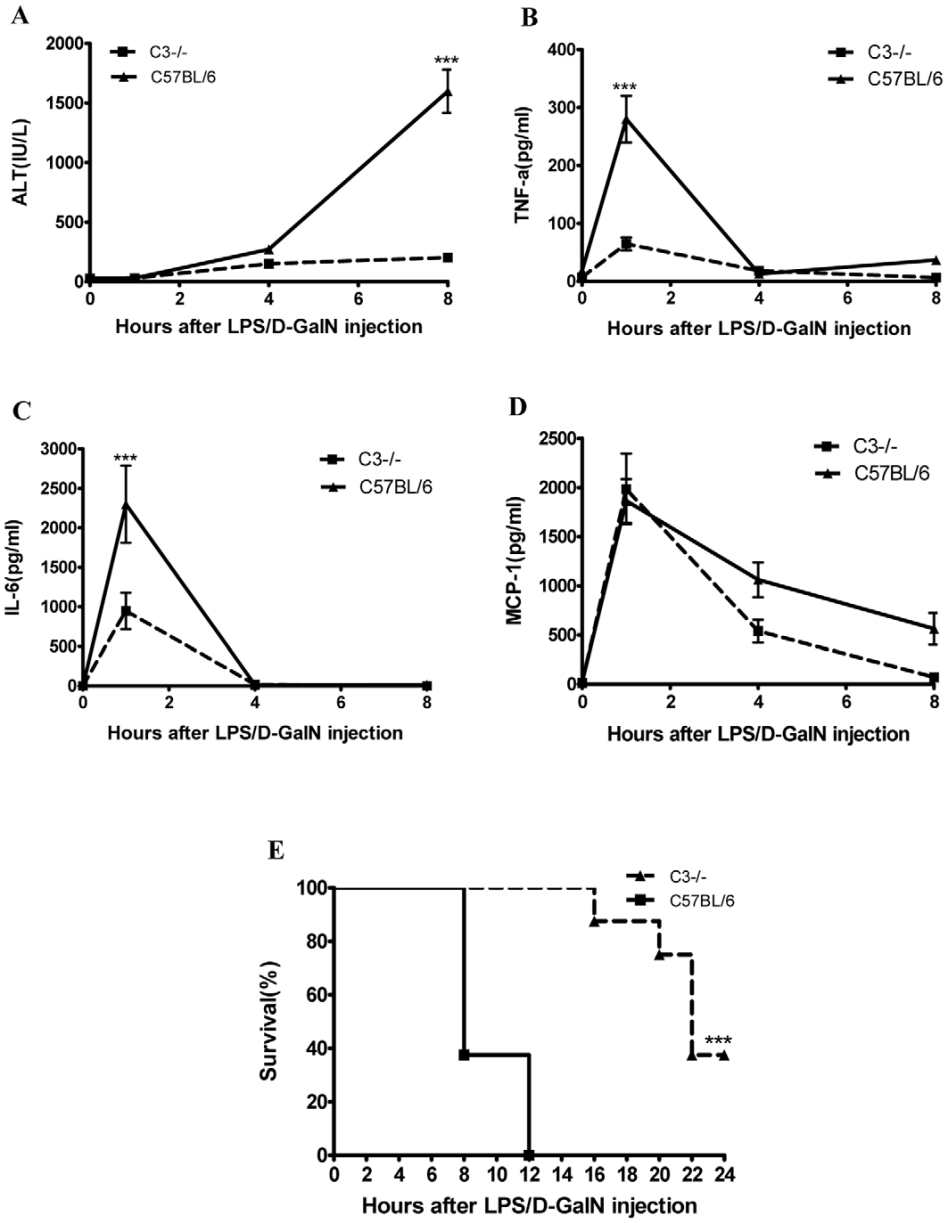
The effects of C3 deficiency on inflammation and survival after LPS/D-GalN injection. (A) The response patterns to different ALT concentrations in C3^−/−^ and wt mice (n = 4–5). (B–D) The serum levels of proinflammatory cytokines after LPS/D-GalN injection. The concentrations of TNF-α and IL-6 in the C3^−/−^ mice were lower than in the wt C57BL/6 mice at 1 hour, but the levels of these cytokines in both groups decreased to the same concentrations at later time points. There was no difference in MCP-1 between the groups (n = 4–5). (E) The C3^−/−^ mice had prolonged life spans and an increased survival rate after LPS/D-GalN injection (n = 8). *** indicate *p*<0.001, in the comparison between the C3^−/−^ and C57BL/6 groups. The means±SEM are shown. The results are representative of 3 separate experiments.

C3 activation is the central step in the complement cascade; therefore, we investigated the effects of C3 deficiency on LPS/D-GalN-induced liver injury. As shown in Fig. 2, the C3^−/−^ mice displayed a significantly lower degree of liver injury after the LPS/D-GalN treatment compared with the wt mice. A detailed examination of whole livers and the H&E staining of mouse liver sections 8 hours after the LPS/D-GalN injection indicated a much lower degree of hemorrhage with less severe vascular congestion, hepatocellular damage and inflammatory cell infiltration in the C3^−/−^ mice (Fig. 2G–J and L) compared to the wt mice (Fig. 2A–D and F). The deposition of C5b-9 was detected in wt mice but no C5b-9 deposition in C3^−/−^ mice (Fig. 2E and K). The serum ALT concentrations were also significantly lower in the C3^−/−^ mice compared with the wt mice from 4 to 8 hours after the LPS/D-GalN injection (Fig. 3A). The serum levels of TNF-α and IL-6 were significantly lower in the C3^−/−^ mice than in the wt mice at 1 hour after the LPS/D-GalN injection, returning to baseline in both groups by 4 hours (Fig. 3B and C). There was no significant difference in the MCP-1 levels between the C3^−/−^ and wt mice after the LPS/D-GalN injection (Fig. 3D). We also found no differences in the serum levels of IL-10, IFN-γ or IL-12p70 between the C3^−/−^ and wt mice after the LPS/D-GalN injection (data not shown). Notably, C3−/− mice demonstrated a significantly improved survival rate after LPS/D-GalN injection compared with the wt mice, with approximately 30% of the C3^−/−^ mice surviving for 24 hours compared with a 100% mortality rate for wt mice by 12 hours after the LPS/D-GalN challenge (*P*<0.001, n = 8) (Fig. 3E). All mice surviving for 24 hours recovered from any signs of morbidity and appeared healthy. Taken together, these data indicate that C3^−/−^ deficiency provides protection against liver injury induced by LPS/D-GalN.

### Inhibition of C3aR signaling suppresses liver inflammation and attenuates the LPS-D/GalN-induced liver damage

**Figure 4:**
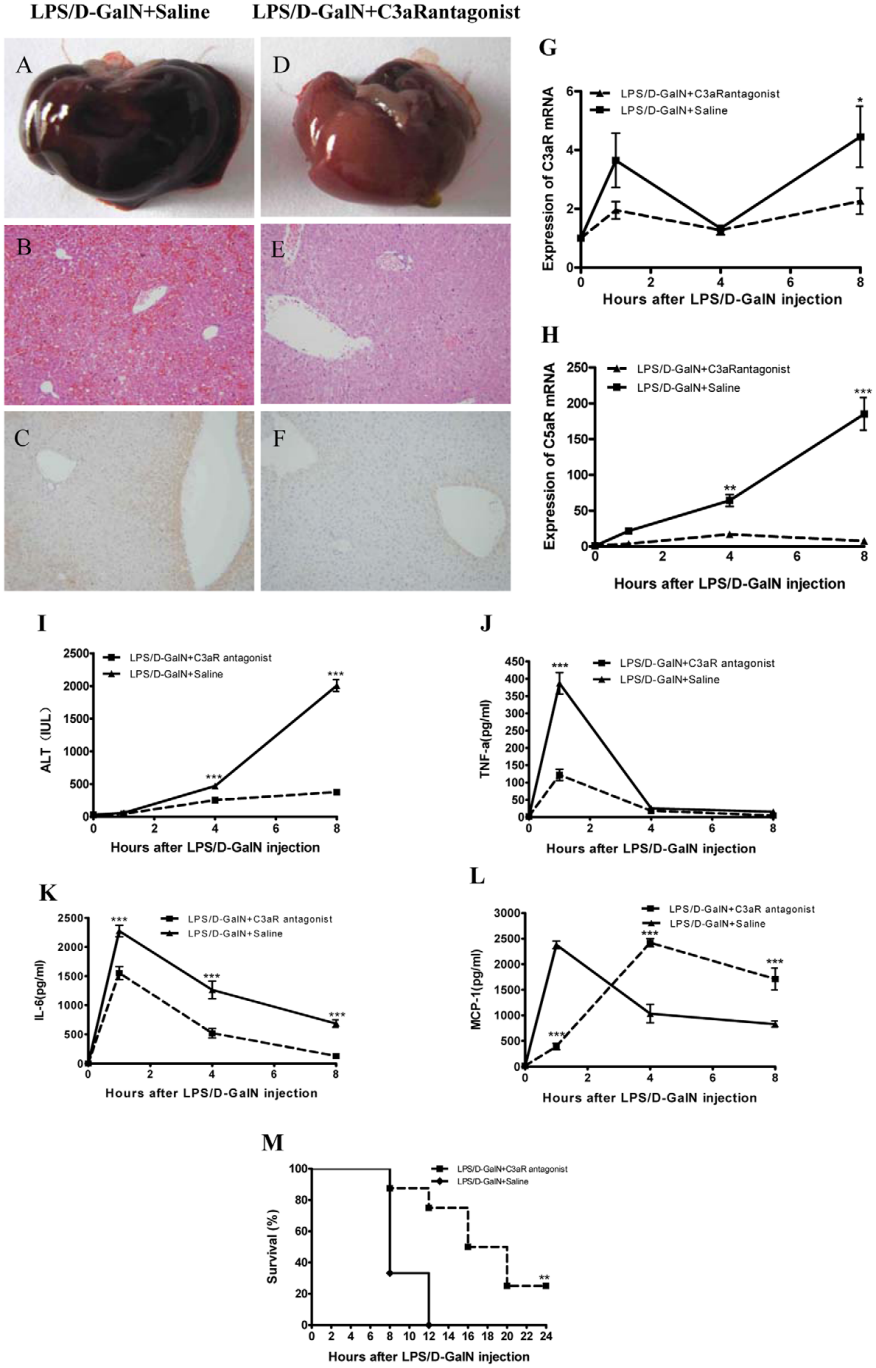
Alleviation of the liver injuries in mice after treatment with C3aR antagonist. (A–F) C3aR antagonist group mice displayed reduced liver damage (A–B, D–E) and decreased C3 deposition (C, F) 8 hours after LPS/D-GalN injection. (G–H) *C3aR* mRNA expression decreased at 8 hours compared with that of the saline group, whereas *C5aR* mRNA expression decreased from 4 to 8 hours (n = 4–5). (I) The different response patterns of ALT concentration in the C3aR antagonist group mice and the wt mice (n = 4–5). (J–L) The concentrations of TNF-α and IL-6 in the C3aR antagonist mice were lower than in the saline group. There was a delayed increase in MCP-1 in the C3aR antagonist group (n = 4–5). (M) Treatment with the C3aR antagonist increased the survival rate of the mice after LPS/D-GalN injection (n = 8). *, **and *** indicate *p*<0.05, *p*<0.01 and *p*<0.001, respectively, relative to the saline group. The means±SEM are shown. Magnification of the H&E and immunohistochemically stained images: ×200. The results are representative of 3 separate experiments.

As shown in Fig. 1, the mRNA expression of both *C3aR* and *C5aR* was upregulated following the LPS/D-GalN-induced liver injury. The complement activation products C3a and C5a are known candidates for mediating liver damage. Therefore, we investigated the effects of C3aR inhibition on liver inflammation and injury. A gross examination of livers and a microscopic analysis of liver sections from mice 8 hours after the LPS/D-GalN injection revealed that the treatment with the C3aR antagonist markedly reduced the amount of liver hemorrhage and parenchymal damage compared with the saline group mice after the LPS/D-GalN injection (Fig. 4A, B, D and E). This reduced injury was associated with less C3 deposition in the liver tissues of the C3aR antagonist-treated mice compared with the control group (Fig. 4C and F). Blocking the interaction of C3a with C3aR also reduced the *C3aR* mRNA and *C5aR* mRNA levels compared with the saline group and resulted in a more significant reduction in the *C5aR* mRNA compared with that of *C3aR* mRNA (Fig. 4G and H). Furthermore, the ALT levels were also significantly lower in the C3aR antagonist-treated mice compared with the saline group from 4 to 8 hours post-LPS/D-GalN injection (Fig. 4I). The serum levels of TNF-α, IL-6 and MCP-1 were reduced in the C3aR antagonist-treated mice at the early time points after LPS/D-GalN injection; however, 4 hours later, the MCP-1 level had increased in the C3aR antagonist-treated mice (Fig. 4J–L). Finally, C3aR blockage improved the survival rate of the LPS/D-GalN-injected mice (*P*<0.01, n = 8) (Fig. 4M).

### Inhibition of C5aR signaling and targeted complement inhibition alleviates liver injury in LPS/D-GalN-injected mice

**Figure 5:**
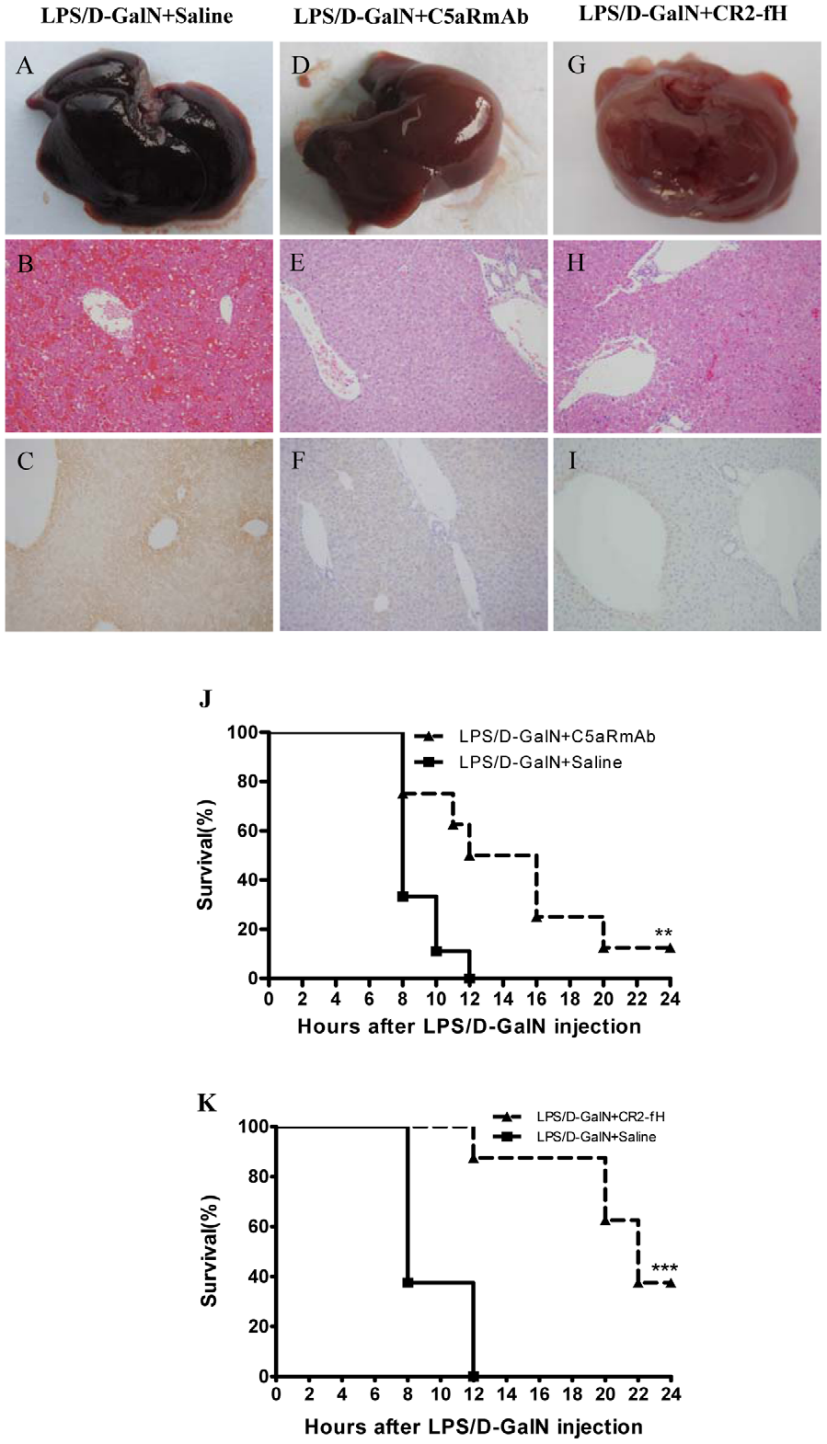
Inhibition of C5aR signaling and targeted complement inhibition alleviates liver injury. (A–I) Both C5aRmAb and CR2-fH groups displayed reduced liver damage (A–B, D–E, G–H) and decreased C3 deposition (C, F, I) 8 hours after LPS/D-GalN injection. (J) The different response patterns of ALT concentration in the C3aR antagonist mice and the wt mice (n = 4–5). (J–K) Treatment with the C5aR antagonist or CR2-fH increased the survival rate of the mice after LPS/D-GalN injection (n = 8). ** and *** indicate *p*<0.01 and *p*<0.001, respectively, relative to the saline group. The means±SEM are shown. Magnification of the H&E and immunohistochemically stained images: ×200. The results are representative of 3 separate experiments.

The effects of C5aR inhibition on liver inflammation and injury were also studied. The results were similar to that of C3aR antagonist treatment with attenuated liver hemorrhage and parenchymal damage, decreased C3 deposition compared with the control mice (Fig. 5A–F), and improved the survival rate (*P*<0.01, n = 8) (Fig. 5J). These results indicated an important role for C5a in mediating LPS/D-GalN-induced liver injury in mice.

Furthermore, the role of the alternative complement pathway was investigated in our injury model using CR2-fH, a complement inhibitor that targets the sites of complement activation and specifically inhibits the alternative pathway [26]. Mice were treated with 0.8 mg CR2-fH i.v. immediately after LPS/D-GalN administration, with saline used as the control treatment. After 8 hours, the CR2-fH-treated mice displayed minimal evidence of focal hemorrhage and parenchymal damage compared with the control group (Fig. 5G and H). C3 deposition was also significantly reduced in the CR2-fH-treated mice compared with the control group (Fig. 5I). Almost 50% of the CR2-fH-treated mice survived for 24 hours, whereas all the control group mice died by 12 hours post-LPS/D-GalN injection (*p*<0.001, n = 8) (Fig. 5K). These data indicate a key role for the alternative complement pathway in causing liver injury in this model.

## Discussion

Acute liver injury is a dramatic clinical syndrome with a high mortality rate and is characterized by sudden and severe damage to hepatic cells, leading to multiorgan failure [29], [30]. Inflammatory cytokines, especially TNF-α, have been linked to the pathogenesis of hepatocyte apoptosis and liver injury [8]–[10]. Recent evidence has indicated a role for complement in the pathogenesis of a variety of liver diseases [13], [31], [32]. Immunohistochemistry analyses performed in patients with fulminant and acute hepatitis have shown that the membrane attack complex (MAC) is deposited around necrotic areas, indicating activation of the complement system and its involvement in the pathogenesis of liver injury [13]. In a hemorrhagic shock and tissue trauma (HS/T) mouse model, complement factor 3 deficiency or temporary C3 depletion by CVF led to both reduced transaminase levels and a blunted cytokine release, with a well-preserved hepatic structure [31]. In a previous study by Stephen Tomlinson [16] in an IRI mouse model, complement was found to play a key role in the enhanced susceptibility of steatotic livers to IRI. The results of these studies led us to hypothesize that the activation of complement or its activation products may mediate hepatic injury in FHF.

LPS is the main pathogenic factor of gram-negative bacteria and can cause systemic inflammatory response syndrome, which may lead to FHF and multiorgan failure. LPS/D-GalN-treated mouse models are frequently used to study the pathogenesis of liver injury [8], [9], [10]. In this study, the data from the mouse model with FHF induced by LPS/D-GalN demonstrated that liver injury was associated with elevated serum C3a levels, extensive hepatic deposition of C3 and increased hepatic expression of *C3aR* mRNA and *C5aR* mRNA, along with elevated serum levels of proinflammatory cytokines and ALT. However, all of the measured parameters of inflammation and injury were significantly reduced by C3 deficiency, blockage of C3aR or inhibition of the alternative complement activation pathway. These data suggest an important role for complement activation, especially the alternative complement activation pathway, in the pathogenesis of LPS/D-GalN-induced liver injury.

The anaphylatoxins C3a and C5a are proinflammatory polypeptides generated by the activation of the complement cascade. Both C3a and C5a have multifunctional roles in liver inflammation and regeneration via interactions with their cognate receptors, C3aR and C5aR, respectively. C5aR has been shown to be constitutively expressed in Kupffer cells and stellate cells under normal conditions, but its expression is increased in hepatocytes in response to inflammatory cytokines [33], [34], [35], [36]. To identify the potential roles of complement activation products in the production of inflammatory cytokines in mice challenged with LPS/D-GalN, we investigated the effect of C3aR or C5aR blockage respectively on LPS/D-GalN-induced liver inflammation. Blocking the binding of C3a and C5a to their receptors protected mice against liver injury apparently. Furthermore, C3aR antagonist reduced the levels of the inflammatory cytokines TNF-α and IL-6, which suggests that the induction of inflammatory cytokines in our model was dependent on complement. These data are in agreement with previous in vitro findings [37], [38] that C3a and C5a enhanced the release of IL-6 and TNF-α in a dose-dependent and PGE_2_-independent manner and that C5aR antagonist treatment after partial hepatic ischemia and reperfusion significantly decreased serum and tissue TNF-α level and attenuated liver histopathology [39].

Although C3a is generally a less potent mediator than C5a, the serum concentration of C3 is 10 times higher than that of C5 [40]. C3a also regulates vasodilation, increases the permeability of small blood vessels, and induces contraction of smooth muscles. Besides, C3a triggers oxidative burst in macrophages, neutrophils and eosinophils [41], [42], [43], and regulates the synthesis of IL-6 and TNF-α from B cells and monocytes [37]. Studies have found that blockade or knockout of C3aR effectively attenuated tissue damage [44], [45], [46], [47] and C3aR^−/−^ mice are more susceptible than wt mice to an i.v. challenge with LPS [48]. In a rat model of ARDS, C3a had profound systemic hemodynamic and systemic cytokine effects, whereas, C5a exerted direct intra-lung modulation of neutrophil infiltration and cytokine production [49]. In our previous study on acute lung injury induced by paraquat in a mouse model, C3aR inhibition significantly attenuated lung injury and increased the survival rate after paraquat administration [25]. Collectively, the blockade of C3a receptor in this study is capable of offering a significant survival benefit by decreasing the inflammatory response. Also, we found that the blockade of both C3a and C5a signals had similar level of protection (data not shown).

Along with the inflammatory function of complement activation, its potential role in liver regeneration has been recently investigated [50], [51]. He S et al. [52] found that in a combined model of IRI and PHx, both C3 deficiency and high-dose CR2-Crry, an inhibitor of C3 activation, produced severe hepatic injury and high mortality, whereas low-dose CR2-Crry was protective and strengthened hepatic proliferative responses. Taken together, these data confirm the multiple roles of complement activation in liver diseases and indicate that complement activation may have an important role in the pathogenesis of liver injury in our mouse model of FHF induced by LPS-D/GalN.

In our model, increased *C3aR* mRNA expression displayed a two-wave pattern, whereas *C5aR* mRNA expression increased progressively following LPS/D-GalN injection, suggesting that C3a/C3aR and C5a/C5aR signaling may have different functions at different stages of liver injury progression. The first phase of complement activation may be related to the priming of liver cells shortly after LPS-D/GalN injection [36]. Our results show that, compared with the *C3aR* expression trend, the C3aR antagonist significantly downregulated *C5aR* expression following LPS/D-GalN injection, which indicates that C5a may also contribute to LPS-induced liver injury. C5a has generally been regarded as a more potent proinflammatory mediator than C3a. Previous studies have shown that C5a stimulated the synthesis of proinflammatory cytokines and enhanced the transcription of type II acute phase protein α_2_-macroglobulin in the liver [34], [53]. Therefore, our data showing a sharply decreased expression of C5aR further indicate the crucial function of C5a in mediating liver damage in LPS-D/GalN-induced FHF.

In summary, we demonstrated an essential role for complement activation, especially the alternative activation pathway, in the pathogenesis of LPS/D-GalN-induced liver injury. The interaction of anaphylatoxins with their receptors plays a role in the hepatic damage in this model. Inhibiting complement activation represents a potential therapeutic approach for the adjunctive treatment of LPS-induced fulminant hepatic failure.

## References

[1] O'Grady JG, Schalm SW, Williams R (1993). Acute liver failure: redefining the syndromes.. Lancet.

[2] Bhaduri BR, Mieli-Vergani G (1996). Fulminant hepatic failure: pediatric aspects.. Semin Liver Dis.

[3] Bernal W, Auzinger G, Dhawan A, Wendon J (2010). Acute liver failure.. Lancet.

[4] Sass G, Heinlein S, Agli A, Bang R, Schumann J (2002). Cytokine expression in three mouse models of experimental hepatitis.. Cytokine.

[5] Galanos C, Freudenberg MA, Reutter W (1979). Galactosamine-induced sensitization to the lethal effects of endotoxin.. Proc Natl Acad Sci U S A.

[6] Fukuda T, Mogami A, Tanaka H, Yoshikawa T, Hisadome M (2006). Y-40138, a multiple cytokine production modulator, protects against D-galactosamine and lipopolysaccharide-induced hepatitis.. Life Sci.

[7] Tiegs G, Wolter M, Wendel A (1989). Tumor necrosis factor is a terminal mediator in galactosamine/endotoxin-induced hepatitis in mice.. Biochem Pharmacol.

[8] Kudo H, Takahara T, Yata Y, Kawai K, Zhang W (2009). Lipopolysaccharide triggered TNF-alpha-induced hepatocyte apoptosis in a murine non-alcoholic steatohepatitis model.. J Hepatol.

[9] Kuhla A, Eipel C, Abshagen K, Siebert N, Menger MD (2009). Role of the perforin/granzyme cell death pathway in D-Gal/LPS-induced inflammatory liver injury.. Am J Physiol Gastrointest Liver Physiol.

[10] Ikeda T, Abe K, Kuroda N, Kida Y, Inoue H (2008). The inhibition of apoptosis by glycyrrhizin in hepatic injury induced by injection of lipopolysaccharide/D-galactosamine in mice.. Arch Histol Cytol.

[11] Markiewski MM, Lambris JD (2007). The role of complement in inflammatory diseases from behind the scenes into the spotlight.. Am J Pathol.

[12] Hillebrandt S, Wasmuth HE, Weiskirchen R, Hellerbrand C, Keppeler H (2005). Complement factor 5 is a quantitative trait gene that modifies liver fibrogenesis in mice and humans.. Nat Genet.

[13] Pham BN, Mosnier JF, Durand F, Scoazec JY, Chazouilleres O (1995). Immunostaining for membrane attack complex of complement is related to cell necrosis in fulminant and acute hepatitis.. Gastroenterology.

[14] Bykov I, Junnikkala S, Pekna M, Lindros KO, Meri S (2006). Complement C3 contributes to ethanol-induced liver steatosis in mice.. Ann Med.

[15] Roychowdhury S, McMullen MR, Pritchard MT, Hise AG, van Rooijen N (2009). An early complement-dependent and TLR-4-independent phase in the pathogenesis of ethanol-induced liver injury in mice.. Hepatology.

[16] He S, Atkinson C, Evans Z, Ellett JD, Southwood M (2009). A role for complement in the enhanced susceptibility of steatotic livers to ischemia and reperfusion injury.. J Immunol.

[17] Ward PA (2004). The dark side of C5a in sepsis.. Nat Rev Immunol.

[18] Guo RF, Ward PA (2005). Role of C5a in inflammatory responses.. Annu Rev Immunol.

[19] Guo RF, Riedemann NC, Ward PA (2004). Role of C5a-C5aR interaction in sepsis.. Shock.

[20] Croner RS, Lehmann TG, Fallsehr C, Herfarth C, Klar E (2004). C1-inhibitor reduces hepatic leukocyte-endothelial interaction and the expression of VCAM-1 in LPS-induced sepsis in the rat.. Microvasc Res.

[21] Zhao L, Ohtaki Y, Yamaguchi K, Matsushita M, Fujita T (2002). LPS-induced platelet response and rapid shock in mice: contribution of O-antigen region of LPS and involvement of the lectin pathway of the complement system.. Blood.

[22] Schmid RA, Zollinger A, Singer T, Hillinger S, Leon-Wyss JR (1998). Effect of soluble complement receptor type 1 on reperfusion edema and neutrophil migration after lung allotransplantation in swine.. J Thorac Cardiovasc Surg.

[23] Huang Y, Qiao F, Atkinson C, Holers VM, Tomlinson S (2008). A novel targeted inhibitor of the alternative pathway of complement and its therapeutic application in ischemia/reperfusion injury.. J Immunol.

[24] Rynkowski1 MichalA, Kim1 GraceH, Garrett MatthewC, Zacharia BradE, Otten MarcL (2009). C3a receptor antagonist attenuates brain injury after intracerebral hemorrhage.. Journal of Cerebral Blood Flow & Metabolism.

[25] Sun Shihui, Wang Hanbin, Zhao Guangyu, An Yingbo, Guo Yan (2011). Complement Inhibition Alleviates Paraquat-Induced Acute Lung Injury.. AJRCMB.

[26] Huang Yuxiang, Qiao Fei, Atkinson Carl, Holers VMichael, Tomlinson Stephen (2008). A Novel Targeted Inhibitor of the Alternative Pathway of Complement and Its Therapeutic Application in Ischemia/Reperfusion Injury.. J Immunol.

[27] De Steenwinkel JE, De Knegt GJ, Ten Kate MT, Van Belkum A, Verbrugh HA (2009). Immunological parameters to define infection progression and therapy response in a well-defined tuberculosis model in mice.. Int J Immunopathol Pharmacol.

[28] Livak KJ, Schmittgen TD (2001). Analysis of relative gene expression data using real-time quantitative PCR and the 2(−Delta Delta C(T)) Method.. Methods.

[29] Tunon MJ, Alvarez M, Culebras JM, Gonzalez-Gallego J (2009). An overview of animal models for investigating the pathogenesis and therapeutic strategies in acute hepatic failure.. World J Gastroenterol.

[30] Dejager L, Libert C (2008). Tumor necrosis factor alpha mediates the lethal hepatotoxic effects of poly(I:C) in D-galactosamine-sensitized mice.. Cytokine.

[31] Cai C, Gill R, Eum HA, Cao Z, Loughran PA (2010). Complement factor 3 deficiency attenuates hemorrhagic shock-related hepatic injury and systemic inflammatory response syndrome.. Am J Physiol Regul Integr Comp Physiol.

[32] Tu Z, Li Q, Chou HS, Hsieh CC, Meyerson H (2011). Complement mediated hepatocytes injury in a model of autoantibody induced hepatitis.. Immunobiology.

[33] Schieferdecker HL, Rothermel E, Timmermann A, Gotze O, Jungermann K (1997). Anaphylatoxin C5a receptor mRNA is strongly expressed in Kupffer and stellate cells and weakly in sinusoidal endothelial cells but not in hepatocytes of normal rat liver.. FEBS Lett.

[34] Schieferdecker HL, Schlaf G, Jungermann K, Gotze O (2001). Functions of anaphylatoxin C5a in rat liver: direct and indirect actions on nonparenchymal and parenchymal cells.. Int Immunopharmacol.

[35] Koleva M, Schlaf G, Landmann R, Gotze O, Jungermann K (2002). Induction of anaphylatoxin C5a receptors in rat hepatocytes by lipopolysaccharide in vivo: mediation by interleukin-6 from Kupffer cells.. Gastroenterology.

[36] Markiewski MM, Mastellos D, Tudoran R, DeAngelis RA, Strey CW (2004). C3a and C3b activation products of the third component of complement (C3) are critical for normal liver recovery after toxic injury.. J Immunol.

[37] Fischer WH, Jagels MA, Hugli TE (1999). Regulation of IL-6 synthesis in human peripheral blood mononuclear cells by C3a and C3a(desArg).. J Immunol.

[38] Cavaillon JM, Fitting C, Haeffner-Cavaillon N (1990). Recombinant C5a enhances interleukin 1 and tumor necrosis factor release by lipopolysaccharide-stimulated monocytes and macrophages.. Eur J Immunol.

[39] Arumugam TV, Woodruff TM, Stocks SZ, Proctor LM, Pollitt S (2004). Protective effect of a human C5a receptor antagonist against hepatic ischaemia-reperfusion injury in rats.. J Hepatol.

[40] Abe M Complement activation and inflammation.. Rinsho Byori 2006.

[41] Murakami Y, Imamichi T, Nagasawa S (1993). Characterization of C3a anaphylatoxin receptor on guinea-pig macrophages.. Immunology.

[42] Elsner J, Oppermann M, Czech W, Kapp A (1994b). C3a activates the respiratory burst in human polymorphonuclear neutrophilic leukocytes via pertussis toxinsensitive G-proteins.. Blood.

[43] Elsner J, Oppermann M, Czech W, Dobos G, Schopf E (1994a). C3a activates reactive oxygen radical species production and intracellular calcium transients in human eosinophils.. Eur J Immunol.

[44] Mizutani N, Nabe T, Yoshino S (2009). Complement C3a regulates late asthmatic response and airway hyperresponsiveness in mice.. J Immunol.

[45] Garrett MC, Otten ML, Starke RM, Komotar RJ, Magotti P (2009). Synergistic neuroprotective effects of C3a and C5a receptor blockade following intracerebral hemorrhage.. Brain Res.

[46] Ducruet AF, Hassid BG, Mack WJ, Sosunov SA, Otten ML (2008). C3a receptor modulation of granulocyte infiltration after murine focal cerebral ischemia is reperfusion dependent.. J Cereb Blood Flow Metab.

[47] Bao L, Osawe I, Haas M, Quigg RJ (2005). Signaling through up-regulated C3a receptor is key to the development of experimental lupus nephritis.. J Immunol.

[48] Bautsch W, Hoymann HG, Zhang Q, Meier-Wiedenbach I, Raschke U (2000). Cutting edge: guinea pigs with a natural C3a-receptor defect exhibit decreased bronchoconstriction in allergic airway disease: evidence for an involvement of the C3a anaphylatoxin in the pathogenesis of asthma.. J Immunol.

[49] Proctor LM, Strachan AJ, Woodruff TM, Mahadevan IB, Williams HM (2006). Complement inhibitors selectively attenuate injury following administration of cobra venom factor to rats.. Int Immunopharmacol.

[50] Mastellos D, Papadimitriou JC, Franchini S, Tsonis PA, Lambris JD (2001). A novel role of complement: mice deficient in the fifth component of complement (C5) exhibit impaired liver regeneration.. J Immunol.

[51] Strey CW, Markiewski M, Mastellos D, Tudoran R, Spruce LA (2003). The proinflammatory mediators C3a and C5a are essential for liver regeneration.. J Exp Med.

[52] He S, Atkinson C, Qiao F, Cianflone K, Chen X (2009). A complement-dependent balance between hepatic ischemia/reperfusion injury and liver regeneration in mice.. J Clin Invest.

[53] Schlaf G, Schmitz M, Rothermel E, Jungermann K, Schieferdecker HL (2003). Expression and induction of anaphylatoxin C5a receptors in the rat liver.. Histol Histopathol.

